# Lysosomal acid lipase: at the crossroads of normal and atherogenic cholesterol metabolism

**DOI:** 10.3389/fcell.2015.00003

**Published:** 2015-02-02

**Authors:** Joshua A. Dubland, Gordon A. Francis

**Affiliations:** Department of Medicine, Centre for Heart Lung Innovation, Providence Health Care Research Institute at St. Paul's Hospital, University of British ColumbiaVancouver, BC, Canada

**Keywords:** lysosomal acid lipase, atherosclerosis, foam cells, oxidized LDL, lysosomal storage disorders, ABCA1, smooth muscle cells, macrophages

## Abstract

Unregulated cellular uptake of apolipoprotein B-containing lipoproteins in the arterial intima leads to the formation of foam cells in atherosclerosis. Lysosomal acid lipase (LAL) plays a crucial role in both lipoprotein lipid catabolism and excess lipid accumulation as it is the primary enzyme that hydrolyzes cholesteryl esters derived from both low density lipoprotein (LDL) and modified forms of LDL. Evidence suggests that as atherosclerosis progresses, accumulation of excess free cholesterol in lysosomes leads to impairment of LAL activity, resulting in accumulation of cholesteryl esters in the lysosome as well as the cytosol in foam cells. Impaired metabolism and release of cholesterol from lysosomes can lead to downstream defects in ATP-binding cassette transporter A1 regulation, needed to offload excess cholesterol from plaque foam cells. This review focuses on the role LAL plays in normal cholesterol metabolism and how the associated changes in its enzymatic activity may ultimately contribute to atherosclerosis progression.

## Introduction

Atherosclerosis is characterized biochemically by the accumulation of excess cholesterol in the artery wall. This process is initiated by migration of low density lipoproteins (LDL) and other apolipoprotein B (apoB)-containing lipoproteins across an injured artery endothelium, where they are retained through a charge-charge interaction with matrix proteoglycans in the subendothelial space (Tabas et al., 2007). Within this space the trapped lipoproteins are subject to modification including oxidation and aggregation, converting them to ligands for scavenger receptors on intimal macrophages and smooth muscle cells (SMCs). Excessive scavenger receptor-mediated uptake of modified lipoproteins leads to accumulation of cytosolic cholesteryl ester (CE) droplets in these cells, giving them a “foamy” appearance microscopically, hence their name foam cells. There is also evidence that at later stages of atherosclerosis, both free cholesterol (FC) and CE droplets accumulate within the lysosome itself (Jerome, 2006). Lysosomal acid lipase (LAL) plays the central role in hydrolyzing lipoprotein CE to generate FC, which after leaving the lysosome can be re-esterified in the endoplasmic reticulum to form cytosolic lipid droplets. If excess FC is retained in the lysosome it can inhibit lysosomal activity including that of LAL. The physiologic role of LAL may therefore lead to eventual inhibition of LAL activity in the presence of excess atherogenic lipoprotein uptake, contributing further to the progression of atherosclerosis.

## Lysosomal acid lipase

### Gene expression and mutation

LAL is the enzyme with primary responsibility for hydrolysis of CE and triglycerides in lipoproteins taken up by receptor-mediated endocytosis. LAL has been called by several names, including lipase A, acid cholesteryl ester hydrolase, acid cholesterol esterase, and acid cholesteryl esterase. LAL is a 46 kDa glycoprotein whose gene *LIPA* is found on chromosome 10q23.2-23.3 (Anderson et al., 1993). Human LAL cDNA encodes a 372-amino acid mature protein and a 27-amino acid signaling sequence (Ameis et al., 1994). After undergoing co-translational glycosylation in the endoplasmic reticulum and attachment of mannose-6-phosphate residues in the Golgi apparatus, LAL is targeted to pre-lysosomal compartments (Sando and Henke, 1982; Sheriff et al., 1995). Mutations of *LIPA* result in complete deficiency of LAL in patients with Wolman disease, which is fatal in the first year of life without LAL replacement, or near-total and non-fatal deficiency known as Cholesteryl Ester Storage Disease (CESD). The most common mutation seen in CESD patients is a splice junction mutation at exon 8 of *LIPA*, which leads to ~3–5% of normally spliced LAL protein and LAL activity (Scott et al., 2013; Stitziel et al., 2013). In addition to having fatty liver and spleen enlargement, individuals with CESD have elevated LDL-Cholesterol, low HDL-Cholesterol, and develop premature atherosclerosis, indicating the critical role LAL plays in cellular cholesterol and lipoprotein metabolism (Bernstein et al., 2013; Grabowski et al., 2014).

### Transcriptional regulation

Several reports have demonstrated an increase in LAL mRNA and protein in the artery wall during the progression of atherosclerosis. Ries et al. (1998) reported that LAL mRNA is increased as human blood monocytes differentiate to macrophages, and that increases in LAL mRNA resulted in higher amounts of LAL enzyme within differentiated macrophages. Therefore, macrophages expressing increased levels of LAL may contribute to the increased LAL activity in the atherosclerotic relative to normal artery wall.

LAL has been shown to be regulated by transcription factors involved in the autophagy pathway. The transcription factor forkhead homeobox type protein O1 (FOXO1) is upregulated in response to nutrient restriction (Sandri, 2012), and has been shown to upregulate *LIPA* in adipocytes as part of the autophagy response (Lettieri Barbato et al., 2013). It has also been reported that the basic helix-loop-helix (bHLH) transcription factor EB (TFEB), described as a master regulator of autophagy and lysosomal biogenesis, targets the *LIPA* gene (Sardiello et al., 2009). When cellular nutrient conditions are plentiful TFEB is phosphorylated by mammalian target of Rapamycin complex 1 (mTORC1) and is located in the cytosol on the lysosomal membrane (Settembre et al., 2012). The reader is directed elsewhere for further information regarding upstream signaling mechanisms of autophagy through mTORC1 (He and Klionsky, 2009; Efeyan et al., 2012; Shanware et al., 2013; Sergin and Razani, 2014). Under conditions of starvation and lysosomal stress TFEB is dephosphorylated and translocates to the nucleus for upregulation of genes involved in the lysosomal-autophagic pathway (Settembre et al., 2013). Recently it has been reported that overexpression of TFEB led to a 2.5-fold increase in *LIPA* mRNA in mouse peritoneal macrophages (Emanuel et al., 2014). Whether such an upregulation occurs in response to physiologic excursions of TFEB expression is not yet known. Translocation of TFEB to the nucleus and a modest (0.2-fold) upregulation of *LIPA* also occurred after a 12 h incubation of peritoneal macrophages with oxidized LDL (oxLDL); the TFEB nuclear localization was reduced at 24 h of oxLDL incubation, however, indicating this response of *LIPA* is likely mild and transient (Emanuel et al., 2014). Heltianu et al. (2011) reported no change in *LIPA* expression in SMCs and a 20% reduction in *LIPA* expression in endothelial cells incubated with oxLDL for 24 h. Further studies are required to confirm the importance and cell specificity of TFEB-dependent upregulation of *LIPA* in response to modified forms of LDL and cellular stress induced by excess cell cholesterol.

It has also been demonstrated by Heltianu et al. (2011) that in some cell types *LIPA* expression increases with liver-X-receptor (LXR) and peroxisome proliferator-activated receptor (PPAR) agonists. *LIPA* is not known to contain an LXR or PPAR response element in its promoter region. Bowden et al. (2011) demonstrated in CESD fibroblasts that the LXR agonist TO901317 failed to correct LDL-CE hydrolysis and cholesterol efflux to apolipoprotein A1 (apoA1), indicating the residual expression of LAL in CESD cells was not increased by the LXR agonist. *LIPA* response to LXR and PPARγ agonists may be cell type specific and involve stimulation of the autophagy pathway. LXR is not a known target of TFEB, whereas peroxisome proliferator-activated receptor gamma coactivator 1-alpha (PGC-1α), which interacts with PPARγ, is a transcriptional target of TFEB (Sardiello et al., 2009).

Our understanding of the transcriptional regulation of *LIPA* in the artery wall during the progression of atherosclerosis is far from complete. Initially it was hypothesized by De Duve (1974) that a deficiency in LAL was the cause of intracellular accumulations of lipids in atherosclerosis. Supporting this hypothesis was the observation of lipid-engorged lysosomes in post-mortem liver tissue samples from Wolman disease patients having complete absence of LAL (Patrick and Lake, 1969; Lake and Patrick, 1970) and premature atherosclerosis seen in the near-total LAL deficiency CESD (Sloan and Fredrickson, 1972). Several groups subsequently reported that LAL activity was increased in atherosclerotic tissue (Brecher et al., 1977; Takano, 1978; Subbiah, 1979). Haley et al. (1980) indicated a 2-fold increase in LAL activity in lipid-laden lysosomes isolated by density centrifugation from atherosclerotic tissue homogenates. Davis et al. (1985) reported that increased LAL activity in human aortic lesions at various stages of disease correlated with increased macrophage infiltration. These studies do not support the LAL deficiency hypothesis of atherosclerosis. It is not yet clear whether increased LAL in atherosclerotic tissue is a result of upregulation of LAL within cells or a higher number of LAL-expressing cells present in the lesion, but potentially both.

### Regulatory role of lysosomally-derived cholesterol

Cholesterol is a component of all cell membranes, and is the precursor for oxysterol, steroid hormone, bile acid, and vitamin D synthesis. FC released by lysosomes from lipoprotein and membrane turnover is an important substrate pool for these metabolites. In addition the lysosomally-derived cholesterol pool has multiple regulatory roles through inhibition of sterol regulatory element binding protein (SREBP) cleavage activating protein (SCAP) in the endoplasmic reticulum, and downregulation of SREBP-dependent new cholesterol synthesis and LDL receptor expression by the cell (Brown and Goldstein, 1997). In this way cholesterol released from lysosomes is a critical regulator of overall cell cholesterol homeostasis. When flux of FC from lysosomes is high, oxysterol levels including 27-hydroxycholesterol are increased and bind to the nuclear receptor liver X receptor (LXR), which activates transcription of several genes involved in the removal of cholesterol from cells and other steps in reverse cholesterol transport (Venkateswaran et al., 2000). One of these genes encodes the ATP-binding cassette transporter A1 (ABCA1), a plasma membrane protein that promotes cholesterol efflux to apoA1, the rate-limiting step in the formation of high density lipoproteins (HDL) (Oram and Heinecke, 2005). The importance of lysosomally-derived cholesterol in, and the inability of increased de novo synthesis of cholesterol (Goldstein et al., 1975; Liscum and Faust, 1987) to correct, ABCA1 expression has been demonstrated in the lysosomal cholesterol storage disorders Niemann Pick Disease type C (NPC) and CESD (Choi et al., 2003; Bowden et al., 2011). In both cases the reduced flux of FC out of lysosomes leads to reduced 27-hydroxycholesterol production and reduced ABCA1 expression, the likely cause of low plasma HDL-C in both these disorders (Garver et al., 2010; Bernstein et al., 2013). Delivery of exogenous oxysterols to NPC fibroblasts (Boadu et al., 2006) and recombinant human LAL to CESD fibroblasts (Bowden et al., 2011) were both able to correct ABCA1 expression and cholesterol efflux to apoA1. This indicates that the rate of release of FC from the lysosome after both LAL-dependent CE hydrolysis and the action of Niemann Pick type C1 (NPC1) protein in the lysosomal membrane is a critical determinant of cholesterol-dependent gene regulation. As hydrolysis of CE derived from both LDL and modified LDL in the lysosome is also likely to be a key regulator of ABCA1 expression in cells in atherosclerotic lesions, a defect in LAL activity could result in impaired ABCA1-dependent cholesterol efflux from macrophage and SMC foam cells.

## Lipoprotein uptake and lipid accumulation

**Figure 1:**
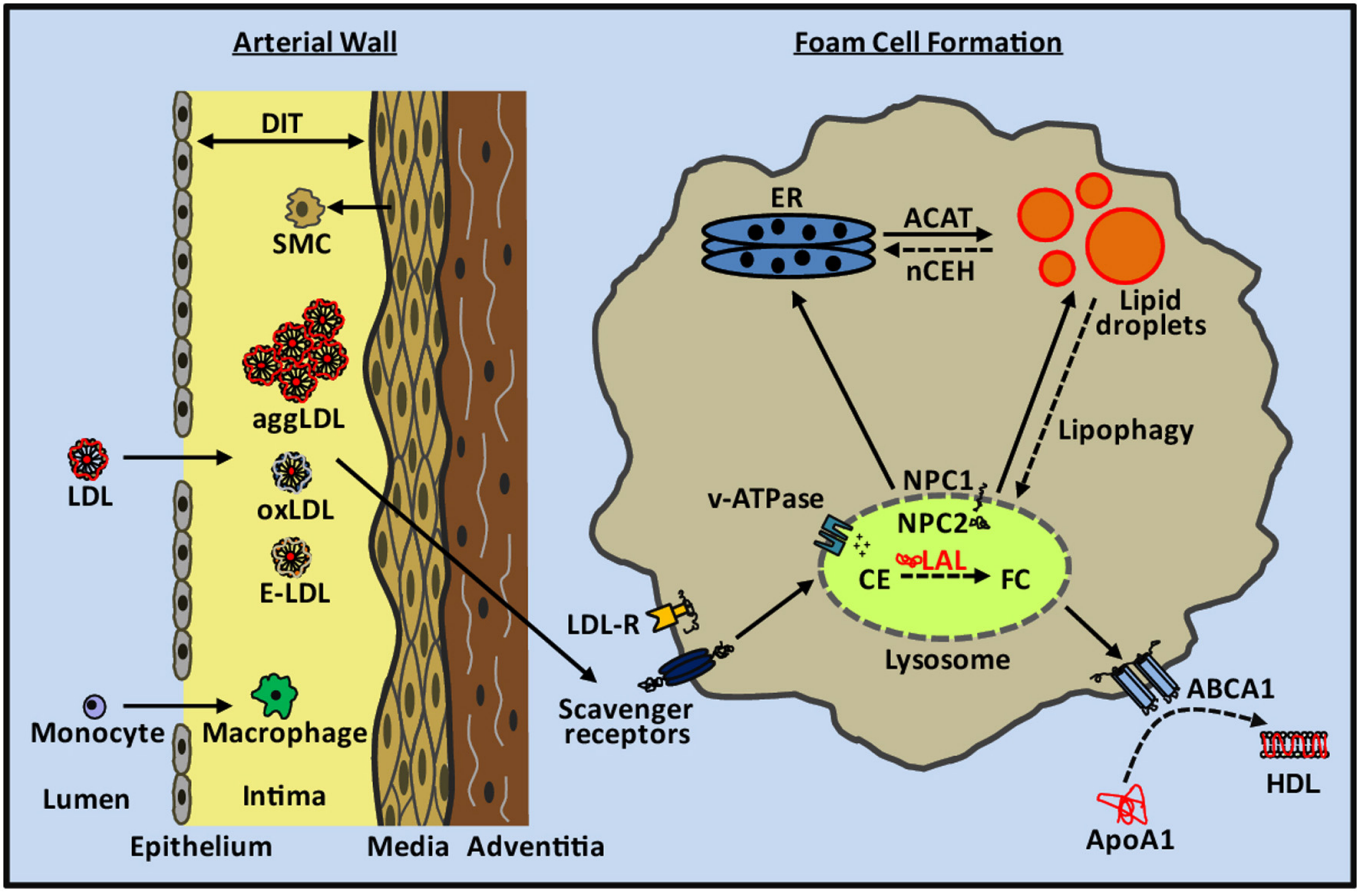
**Cholesterol metabolism in atherosclerotic lesion foam cells**. LDL and other apoB-containing lipoproteins pass through the damaged endothelium and undergo aggregation, oxidation, and enzymatic modification within the intima of the arterial wall. SMCs from the media migrate to the intima and contribute to diffuse intimal thickening (DIT). Monocytes enter through the damaged endothelium and differentiate into macrophages. SMCs and macrophages take up aggLDL, oxLDL, and enzymatically-modified LDL (E-LDL) through scavenger receptors in an unregulated manner, and deliver their cargo to the lysosome through the endocytic pathway. Within the lysosome, lipoprotein cholesteryl esters (CE) are hydrolyzed by LAL to generate free cholesterol (FC). The pH of the lysosome is acidified by the proton pumping action of v-ATPases. FC leaves the lysosome through the concerted action of NPC2 and NPC1 proteins and is transported within the cell including to the endoplasmic reticulum (ER) where it is re-esterified by ACAT and accumulates as lipid droplets within the cytosol. CE in lipid droplets can be hydrolyzed by nCEH and used for cellular functions or removed from the cell along with phospholipids by the actions of ABCA1 to create new HDL particles. CE in lipid droplets can also be transported back to the lysosome through the lipophagy pathway for hydrolysis by LAL and cellular removal of FC via ABCA1. Early atherosclerotic lesion foam cell lipid droplets are primarily cytosolic whereas later stage lesion foam cells contain a mixture of ACAT-derived lipid droplets and lysosomal lipid droplets.

Uptake of LDL through cell surface LDL-receptors is tightly regulated and therefore insufficient to explain intimal foam cell formation (Goldstein and Brown, 1977, 2009). Within the artery wall LDL and other apoB-containing particles become oxidized, aggregated, or enzymatically-modified (Aviram, 1993; Bhakdi et al., 1995; Hoff and Hoppe, 1995; Chisolm and Steinberg, 2000; Torzewski and Lackner, 2006) (Figure 1). These altered forms of LDL are no longer recognized by the LDL receptor but are recognized and taken up by scavenger receptors expressed on macrophages and SMCs, a cellular process that is not feedback regulated and can lead to massive accumulation of cholesterol in these cells (Brown et al., 1979; Brown and Goldstein, 1983; Moore and Freeman, 2006). Scavenger receptors as well as the LDL receptor utilize the endocytic pathway for delivery of cargo to lysosomes. Lipoprotein cholesterol is primarily in the form of CE, which is hydrolyzed within lysosomes by LAL, the only lysosomal lipase known to perform this function (Brown et al., 1975; Goldstein and Brown, 1977; Aviram, 1993; Dhaliwal and Steinbrecher, 2000; Yancey and Jerome, 2001; Griffin et al., 2005). LAL in the lysosome is most catalytically active at pH 3.5–4.5 (Takano et al., 1974; Haley et al., 1980). The FC released from lipoprotein CE is transported out of the lysosome by the concerted actions of the Niemann-Pick Type C2 (NPC2) and NPC1 lysosomal proteins, with one fate of the FC then being re-esterification in the endoplasmic reticulum by acyl-coenzyme A:cholesterol acyltransferase (ACAT) (Kwon et al., 2009). Re-esterification is a protective mechanism to prevent the toxicity of excess FC in membranes (Tabas, 2002), particularly in the endoplasmic reticulum, with the reformed CE then stored in the cytoplasm as benign lipid droplets (Brown and Goldstein, 1983). CE in foam cell cytosolic lipid droplets can be transported back to lysosomes through the autophagic process termed “lipophagy” (Singh et al., 2009); cholesterol derived from this process appears to form a large part of the substrate pool of ABCA1 for efflux from cells (Ouimet et al., 2011).

During early stages of atherosclerosis foam cells appear as visible fatty streaks in the intima of the artery wall (Haust, 1971). In addition to the presence of macrophage derived foam cells, SMCs present in the intima beginning in the pre-atherosclerotic diffuse intimal thickening stage also take up modified forms of LDL via scavenger receptors to become smooth muscle derived foam cells (Li et al., 1995; Doran et al., 2008). It has been previously thought macrophages were the major contributors to foam cells in the intima, however recent studies from our lab suggest SMCs are the source of more than 50% of the total foam cell population in human coronary artery atherosclerotic lesions (Allahverdian et al., 2014).

## Overaccumulation of cholesterol in lysosomes

Retention of LDL and other apoB-containing lipoproteins in the arterial intima by matrix proteoglycans, as a result of charge-charge interactions (Williams and Tabas, 1995, 2005), means these lipoproteins are susceptible to modification to more atherogenic forms. This modification, either by enzymes, oxidants, or through aggregation converts these lipoproteins to ligands for scavenger receptors and allows for the formation of cholesterol-overloaded foam cells (Hoff and Hoppe, 1995; Torzewski and Lackner, 2006). Within the intima LDL has been demonstrated to undergo both extracellular and intracellular modifications (Aviram, 1993; Bhakdi et al., 1995; Hoff and Hoppe, 1995; Torzewski et al., 1998; Chisolm and Steinberg, 2000; Steinberg and Witztum, 2002; Stocker and Keaney, 2004; Wen and Leake, 2007). LAL and lysosomal enzymes have also been implicated in the modification of LDL, and, conversely, modified forms of LDL have been shown to affect lysosomal function and lead to changes in LAL activity.

### Extracellular modification of LDL by LAL

LAL is present within the extracellular space of atherosclerotic intima (Hakala et al., 2003), and there are indications that LAL may actively participate in the modification of retained LDL. Secretion of lysosomal enzymes by macrophages is known to occur without stimuli, and increase under inflammatory conditions (Schnyder and Baggiolini, 1978). Catalytic activity of LAL would require conditions of at least localized reduced pH in the extracellular space. Naghavi et al. (2002) reported that pH heterogeneity exists within atherosclerotic lesions with increased acidity in lipid-rich, macrophage-containing areas compared to calcified areas of human and rabbit atherosclerotic plaque. Micro-environments with much lower pH supportive of LAL activity may exist within lesions, as macrophages can acidify their pericellular space through the action of proton pumps (Tapper and Sundler, 1992) and by secreting lactic acid (Newsholme et al., 1987). Buton et al. (1999) reported that CE present in matrix-retained aggregated LDL (aggLDL) may be hydrolyzed by LAL during extensive cell-surface contact with cultured macrophages. Fluorescence microscopy was utilized to indicate that the matrix-retained aggLDL was present on the cell surface during CE hydrolysis; definitive proof that the observed CE hydrolysis is extracellular is still needed. Thus, LAL might be present in a catalytically active form in the pericellular space of macrophages within lesions, which could contribute to extracellular CE hydrolysis and atherogenic modification of apoB containing particles. In support of CE hydrolysis of LDL increasing the atherogenicity of LDL, human macrophages have been shown to take up LDL modified by hydrolysis with neutral cholesteryl ester hydrolase (nCEH) at a rate exceeding that of oxLDL or acLDL (Bhakdi et al., 1995). Further evidence is required to know whether extracellular hydrolysis of LDL CE by LAL increases uptake of the modified LDL by scavenger receptors.

### Oxidative modification of LDL

Oxidation of LDL is proposed to contribute to foam cell formation by virtue of oxLDL being a ligand for scavenger receptors. The presence of oxLDL within atherosclerotic lesions has been demonstrated by immunostaining using antibodies generated against oxLDL epitopes (Mehrabi et al., 2000). Within the artery wall, oxidation of LDL may occur by a number of concurrent mechanisms involving enzymes (e.g., myeloperoxidase, NADPH oxidase, lipoxygenase, and xanthine oxidase), peroxynitrite-generators, superoxide, low levels of metal ions, and thiols (Gaut and Heinecke, 2001; Parthasarathy et al., 2010; Yoshida and Kisugi, 2010), and is thought to occur mainly within the interstitial fluid of atherosclerotic lesions.

The presence of oxidized sterols within human atherosclerotic tissue and lesion LDL has been reported. Steinbrecher and Lougheed (1992) found 7-ketocholesterol, 7-hydroxycholesterol, and 5,6-epoxycholesterol in LDL isolated from atherosclerotic but not healthy aortas. Significant accumulations of oxysterols are present in atherosclerotic lesions (Brown and Jessup, 1999). Quantitative comparison of *in vitro* studies conducted using oxLDL is difficult due to the variety of methods by which LDL is oxidized and the degree of oxidation induced (Yancey and Jerome, 2001; Orso et al., 2011). Oxidative modification of apoB by oxidized lipids reduces recognition of LDL by the LDL receptor and increases its affinity for scavenger receptors, making oxLDL a potent promoter of foam cell formation *in vitro* (Jessup and Kritharides, 2000). *In vitro* studies show a significant fraction of the sterols present in oxLDL are retained within the lysosome and that oxidized CE are resistant to hydrolysis by LAL (Yancey and Jerome, 1998; Brown et al., 2000). Of note, LDL isolated from human atherosclerotic plaques was found to be largely aggregated and only mildly oxidized (Steinbrecher and Lougheed, 1992; Niu et al., 1999).

### Aggregation of LDL

**Figure 2:**
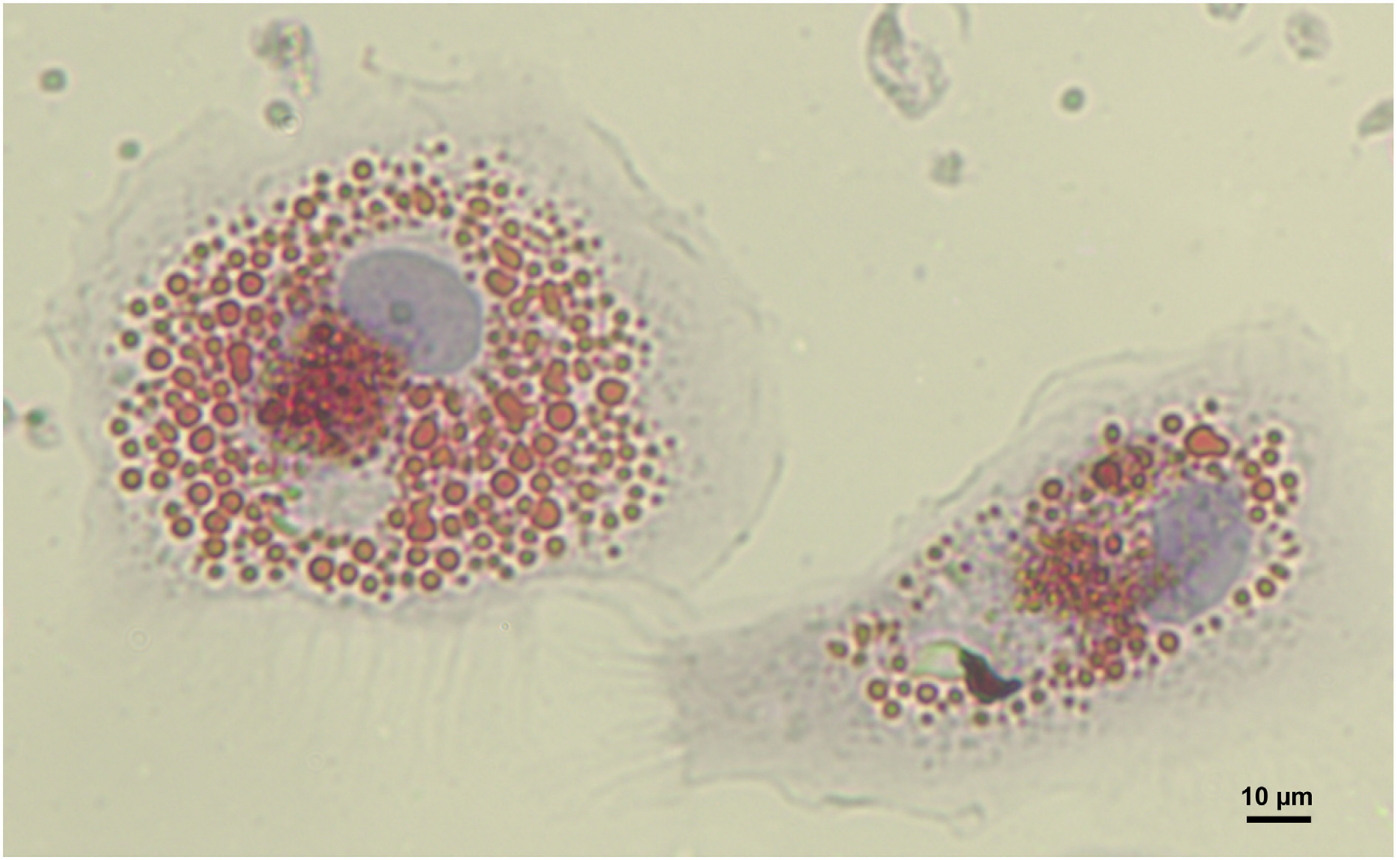
**Human monocyte derived macrophage foam cells**. Foam cells were generated by incubation of macrophages with 100 μg/mL aggregated LDL for 48 h. Cells were then fixed and intracellular lipids stained using Oil Red O (image provided by Dubland, J. A.).

Isolated aortic LDL fractions from human lesions are potent inducers of macrophage CE accumulation relative to normal aortic LDL fractions and plasma LDL (Goldstein et al., 1981; Hoff and Morton, 1985). Steinbrecher and Lougheed (1992) demonstrated that this affect was due primarily to aggLDL isolated from human atheromas. The presence of aggLDL in the extracellular space of human atherosclerotic tissue was also reported using electron microscopy (Guyton and Klemp, 1993). *In vivo*, aggregation of LDL may be a result of a variety of stimuli including oxidation, extracellular matrix interactions, phospholipase A_2_ and C, sphingomyelinase, and glycosylation (Falcone and Salisbury, 1988; Suits et al., 1989; Hoff et al., 1990; Xu and Tabas, 1991; Tertov et al., 1992; Maor and Aviram, 1999; Xu and Lin, 2001; Kruth, 2002). As oxidation is known to cause aggregation of LDL it may be difficult to separate the relative contributions of oxLDL and aggLDL to foam cell (Figure 2) formation in the atherosclerotic intima.

## Effects of excess modified LDL uptake on lysosomal hydrolytic function

Previous studies have reported variable effects of different forms of modified LDL on accumulation of cholesterol either in the cytosol alone or also in lysosomes, with oxLDL and aggLDL causing accumulation of cholesterol in lysosomes, while enzymatically-modified and acetylated LDL cause mainly cytosolic accumulations (Yancey and Jerome, 2001; Griffin et al., 2005; Orso et al., 2011). Treatment of THP-1 macrophages in culture with mildly oxLDL or aggLDL shows similar trends in loss of lysosomal hydrolysis (Yancey and Jerome, 2001; Griffin et al., 2005). Initially CE hydrolysis is not inhibited but lysosomal sequestration of FC occurs. After prolonged exposure to mildly oxLDL or aggLDL (48 h or more) increasing inhibition of CE hydrolysis occurs and lysosomal accumulation switches to mainly CE (Yancey and Jerome, 2001; Griffin et al., 2005).

Some variability between species has been observed (Griffin et al., 2005). Yancey and Jerome (1998) reported that THP-1 human macrophages and pigeon macrophages store FC and CE derived from mildly oxLDL within lysosomal compartments, whereas mouse macrophages stored most of the cholesterol in cytosolic lipid droplets. Interestingly, acLDL was efficiently hydrolyzed in macrophages regardless of the species and led to cytosolic lipid droplet accumulation (Yancey and Jerome, 1998). Maor and Aviram (1994) reported that the addition of oxysterols isolated from oxLDL to medium when loading J-774 mouse macrophages with acLDL led to significant lysosomal FC accumulations.

Cox et al. (2007) have reported a general loss of lysosomal function including reduction in LAL-dependent CE hydrolysis over time following incubation of THP-1 human macrophages with mildly oxLDL or aggLDL. The similar effects using unoxidized aggLDL and oxLDL suggest this inhibition of lysosomal function is not specific to the effects of oxidized lipids. Increasing levels of lysosomal membrane FC during lipid loading were shown to inhibit lysosomal acidification (Cox et al., 2007). It was demonstrated using fluorescence quenching studies that excess FC in the lysosomal membrane leads to loss of acidity as a result of inhibition of vacuolar H^+^-ATPase (v-ATPase) proton pumping activity in the lysosomal membrane, without a change in the amount of v-ATPase protein. The resultant increase in pH within the lysosome corresponded with a time-dependent inhibition of CE hydrolysis and accumulation of apoB, indicating a general decline in lysosomal hydrolytic activity. After 7 days of incubation with mildly oxLDL or aggLDL, the majority of lysosomal vesicles within THP-1 macrophages had pH >4.8. Vacuolar ATPase activity could be partially recovered if isolated lysosomes were treated with cyclodextrins in order to remove membrane cholesterol. As LAL is only active at acidic pH, with maximal activity ~pH 4 and very little activity above pH 4.5 (Burton et al., 1980; Haley et al., 1980), this loss of v-ATPase activity in response to cholesterol loading of the lysosomal membrane may be the main cause of loss of hydrolytic activity of LAL, and hence lead to lysosomal lipid droplet accumulations.

In contrast to oxLDL and aggLDL, incubation of THP-1 macrophages with acLDL, an experimental rather than physiologic form of modified LDL, did not result in an increase in lysosomal membrane FC or an increase in lysosomal pH (Cox et al., 2007). The reasons for the differential effects of acLDL and oxLDL or aggLDL on sequestration of FC in lysosomal membranes following hydrolysis by LAL are unknown. In order to investigate if the observed neutralization of lysosomal pH and inactivation of LAL following oxLDL uptake could be reversed by acLDL, THP-1 macrophages were incubated for 3 days in the presence of oxLDL to accumulate lysosomal CE and then chase incubated with acLDL for an additional 3 days (Jerome et al., 2008). Hydrolysis of CE in the chase acLDL incubation was inhibited and the loss of acidic pH from the initial loading with oxLDL was maintained, indicating lysosomal dysfunction was not readily reversible. Similar results were found in human monocyte derived macrophages. This data indicates that inhibition of LAL activity is the result of a long-lived alteration in lysosomal function under excess lipid loading.

Emanuel et al. (2014) also found a loss of lysosomal acidity occurred in mouse peritoneal macrophages at 72 h of oxLDL treatment and in isolated macrophages from atherosclerotic aortic tissue of ApoE^−/−^ mice. This group also reported that overexpression of TFEB in mouse peritoneal macrophages was able to preserve lysosomal acidity, by mechanisms that are not yet clear.

An interesting study by Ullery-Ricewick et al. (2009) demonstrated that in THP-1 human macrophages lysosomal CE accumulations from aggLDL could be reduced by 50% by chase incubation with triglyceride (TG)-rich lipid dispersions or VLDL. It was shown that TG treatment reestablished acidic pH by restoring proton pumping by v-ATPases in the lysosomal membrane (Ullery-Ricewick et al., 2009). TGs also increased LAL activity, with no change in LAL expression. The exact mechanism by which TG-containing particles restore lysosomal function in foam cells remains to be determined; however, it was demonstrated to not be a result of competitive uptake of TG-rich vs. CE-rich particles (Ullery-Ricewick et al., 2009). This is further evidence that inhibition of v-ATPase proton pumping in the lysosomal membrane leads to loss of LAL activity and lysosomal CE accumulations, but may be reversible under some conditions. Further investigation is needed to evaluate the role of TGs in lysosomes of arterial wall foam cells, including the signaling role of TG-derived fatty acids on the LXR and PPAR metabolic pathways.

An alternative or additional reason for the increase in lysosomal pH is that excess membrane FC and oxidized FC can cause lysosomal leakiness, leading to a loss of the proton gradient. Li et al. (1998) reported that incubation of J-744 macrophages with oxLDL resulted in leakage of lysosomal enzymes into the cytosol. Yuan et al. (2000) found that a mixture of cholesterol oxidation products found in oxLDL were also able to induce damage to lysosomes and leakage of lysosomal contents into the cytosol, and eventual macrophage cell death. Although leakiness was not implicated in the studies by Cox et al. (2007) using mildly oxLDL or aggLDL, as apoptosis was not observed, it may be a long term contributing factor *in vivo*. The presence of free cholesterol crystals has been reported in J774 macrophage foam cells (Tangirala et al. (1994), and treatment of mouse peritoneal macrophages with free cholesterol was shown to induce lysosomal membrane leakiness (Emanuel et al. (2014). An increase in lysosomal pH has also been shown to increase extracellular excretion of lysosomal enzymes (Tapper and Sundler, 1990), which may contribute to the extracellular LAL observed in atherosclerotic lesions (Hakala et al., 2003). Overall it appears that increases in lysosomal FC induce both defects in lysosome acidification and leakiness of the lysosomal membrane.

Oxidation of apoB-100 has been reported to increase its resistance to degradation by cathepsin D in mouse peritoneal macrophages and its accumulation in lysosomal compartments (Lougheed et al., 1991; Jessup et al., 1992; Roma et al., 1992; Hoppe et al., 1994; Mander et al., 1994). Decreased apoB-100 protein degradation may also limit LAL mediated hydrolysis of the CE lipid core of oxLDL. Oxidized phospholipids in oxLDL can inhibit cathepsin D, which may explain the reduced ability to degrade apoB (O'neil et al., 2003). Oxidized phosphatidylcholine-apoB complexes have been found in human atherosclerotic lesions and also in lysosomes of cultured mouse macrophages incubated with oxLDL (Itabe et al., 2000). Brown et al. (2000) found that oxidized CE from heavily oxidized LDL loading are resistant to hydrolysis by LAL and accumulate in lysosomal compartments. Therefore, oxidative modifications to apoB containing particles may lead to impaired lysosomal clearance for reasons additional to those induced by non-oxidized aggLDL.

## Lysosomal cholesterol accumulation in atherosclerosis

In the absence of genetic deficiency in LAL, *in vivo* studies including electron microscopy have indicated that, in addition to storage of CE droplets in the cytoplasm, there are substantial accumulations of FC and CE within the lysosomes of foam cells (Jerome and Yancey, 2003). This phenomenon has been previously reported for both human (Coltoff-Schiller et al., 1976) and animal (Peters et al., 1972; Shio et al., 1974, 1979; Goldfischer et al., 1975; Fowler et al., 1980; Jerome and Lewis, 1985) atherosclerosis. It has been indicated that as atherosclerosis progresses there is initially an increasing amount of FC retained in lysosomes, and at later disease stages also retention of lyososomal CE (Jerome, 2006). A possible conclusion that can be drawn from this is that there is a general loss of lysosomal function due to cholesterol overload following unregulated cellular uptake of modified forms of LDL within artery wall cells. As LAL hydrolyzes lipoprotein CE, the loss of LAL function due to excess FC within the lysosome contributes to the formation of foam cells and the altered distribution of lipid stores within these cells. Therefore, atherosclerosis has been proposed as a type of lysosomal storage disorder (Miller and Kothari, 1969; Peters et al., 1972; Peters and De Duve, 1974; Shio et al., 1974; Coltoff-Schiller et al., 1976; Jerome and Lewis, 1985).

**Figure 3:**
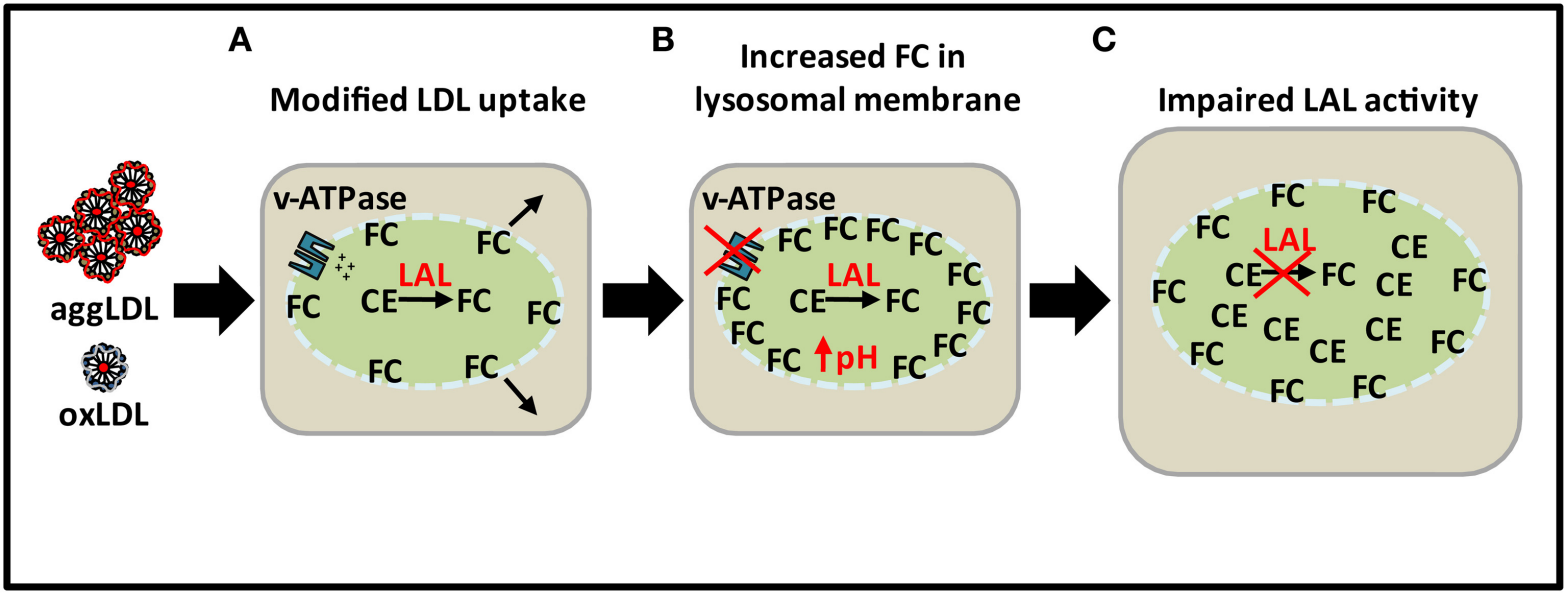
**Loss of lysosomal function with excess lipid loading. (A)** CE from excess modified LDL is hydrolyzed by LAL to produce FC. **(B)** Sequestration of FC in lysosomal membrane leading to increase in pH as a result of inhibition of ATPase proton pumping ability. **(C)** Impairment of LAL activity as a result of increased pH leading to CE accumulation in lysosomes.

As indicated above, the reason for the loss of lysosomal hydrolytic function may not be a result of deficiency in LAL expression as atherosclerosis progresses but rather a decrease in the catalytic activity of LAL as a result of excess lysosomal cholesterol accumulation from modified forms of LDL (Figure 3). LAL remains catalytically active initially but over time FC in the lysosomal membrane increases and inhibits the proton pumping ability of the v-ATPases. As a result, the pH of the lysosome increases and renders LAL catalytically inactivate. Lysosomal CE accumulation then occurs in addition to cytosolic accumulation in later stage atherosclerotic lesions.

Previous studies in atherosclerotic tissue have indicated that LAL activity is increased relative to non-atherosclerotic arteries (Brecher et al., 1977; Takano, 1978; Subbiah, 1979; Haley et al., 1980; Davis et al., 1985). The analytical procedures for those studies, however, involved *ex vivo* acidic pH adjustment and therefore reflects the total amount of potentially active LAL present, but not what may be actually active *in vivo* within intact cells under the influence of excess lipids and altered lysosomal pH. As activity is also reflective of protein amount, these studies may indicate that LAL expression increases to deal with the increased cellular influx of apoB containing particles within the artery wall. It can be proposed that LAL activity and protein amount in arterial cells increases with lipid challenge up to a certain point, either from increased LAL expression per cell or an increase in total LAL-expressing cells, after which the lysosome is overwhelmed with lipids and the activity of LAL decreases due to increased pH caused by excess lysosomal membrane cholesterol and leakiness.

## Indications of a lysosomal cholesterol trafficking defect

The question then arises what is responsible for the elevation of lysosomal FC in response to the excess lipoprotein load in the intima. As NPC1 and NPC2 facilitate the release of FC from the lysosome, investigation of the expression and activity of these proteins may provide useful insight in this area. Previous studies in fibroblasts have suggested that retention of cholesterol within lysosomal compartments may serve to protect cells from endoplasmic reticulum (ER) stress caused by an excess of FC in the ER membrane. Garver et al. (2008) and Jelinek et al. (2009) found that loading normal human fibroblasts with LDL results in reduced SREBP-dependent expression of NPC1 and NPC2. Jelinek et al. (2012) reported that in a mouse model of diet induced obesity, dietary fatty acids but not cholesterol induced down regulation of NPC1 in both hepatic cells and peritoneal fibroblasts via feedback inhibition of the SREBP pathway. In THP-1 macrophages it has been reported that oxLDL treatment for 3 days leads to a buildup of NPC1 protein in the Golgi apparatus (Jerome et al., 2002). Differential responses to cholesterol-dependent manipulations of NPC1 and NPC2 expression have been indicated in the literature. Rigamonti et al. (2005) demonstrated that expression of NPC1 and NPC2 increased in the presence of LXR agonists in human but not mouse macrophages. It has also been demonstrated that treatment of human macrophages with oxLDL leads to increases in NPC1 and NPC2 expression via PPARα- and mitogen-activated protein kinase (MAPK)-dependent pathways (Chinetti-Gbaguidi et al., 2005; Yu et al., 2012). These results indicate that the expression of NPC1 and NPC2 with lipid overload varies between cell types and species. Further investigation is needed to elucidate the reason for FC overload in the lysosomes of atherosclerotic foam cells.

As the rate of release of FC from the lysosome is a regulator of ABCA1 expression, lysosomal dysfunction in atherosclerosis may also lead to decreased expression of ABCA1 in artery wall cells. Choi et al. (2009) have reported that model human intimal SMCs as well as human coronary artery intimal SMCs express lower levels of ABCA1 than medial SMCs. More recently, Allahverdian et al. (2014) reported that intimal SMCs but not myeloid lineage cells express lower levels of ABCA1 in late vs. early stage human atherosclerotic lesions. Further studies are required to determine whether decreased LAL catalytic activity as a result of lysosomal dysfunction is responsible for this defect in ABCA1 expression, and hence further contributing to the overaccumulation of cholesterol in intimal SMC foam cells.

## LAL augmentation in atherosclerosis

Recombinant human LAL (rhLAL) is currently in phase 3 clinical trials to treat LAL deficiency in Wolman disease and CESD. This treatment is potentially life-saving in Wolman disease. Phase 2 clinical trials in patients with CESD using rhLAL demonstrated safety and improvements in serum lipid profiles (Balwani et al., 2013). Long-term follow up will be needed in CESD patients to determine outcomes in terms of cardiovascular events. Several studies in mice have indicated that rhLAL may be beneficial in reversing atherosclerosis. Studies by Du et al. (2001) and Sun et al. (2014) demonstrated that injection of rhLAL into LAL^−/−^ mice was able to reverse the pathogenic storage of lipids in multiple tissues. Du et al. (2004) also showed that administration of repeat doses of rhLAL to LDL-receptor-deficient mice fed a high fat/cholesterol diet resulted in complete regression of early stage lesions in coronary and aortic tissue and a significant reduction in late stage lesions.

Although rhLAL treatment seems to be a viable therapeutic strategy for premature atherosclerosis in CESD patients, it is unclear what the effects would be of rhLAL treatment in patients having atherosclerosis unrelated to a deficiency in LAL. The lysosomal acid lipase A (*LIPA*) gene has been identified as a susceptibility gene for coronary artery disease by several genome-wide association studies (Consortium, 2011; Coronary Artery Disease Genetics, 2011; Wild et al., 2011). For the reasons outlined here, increasing the activity of LAL beyond the normal cellular response may not be an effective strategy. As scavenger receptor mediated uptake of modified forms of LDL in both macrophages and SMCs occurs in atherosclerotic lesions, it is conceivable that rhLAL treatment might increase lysosomal membrane FC in these cells by increasing the rate of lipoprotein CE hydrolysis, and thereby exacerbate lysosomal dysfunction. Reducing the level of atherogenic lipoproteins in plasma and their initial influx into the artery wall, therefore, remains of paramount importance.

## Conclusion

The role of LAL in the progression of atherosclerosis is complex. Modified forms of LDL such as oxLDL and aggLDL lead to lysosomal accumulations of first FC and later CE, indicating an acquired loss of LAL hydrolytic activity. In agreement with this observation, tissue studies have indicated increased lysosomal lipid accumulations in addition to cytosolic lipid droplets at later stages of atherosclerosis. Activity assays of atherosclerotic tissue homogenates have indicated an increase in LAL and also other lysosomal enzymes. As these studies are conducted *ex vivo* using acidic pH adjustment, they are representative of potentially functional LAL present *in vivo* but not necessarily actual LAL activity. Therefore, although the amount of artery wall LAL may increase in response to increased cellular influx of apoB-100 containing lipoproteins, the hydrolytic activity of LAL may decrease over time as lysosomal function is impaired. Sequestration of cholesterol in the lysosomal membrane has been implicated as a cause of lysosomal dysfunction, specifically through the inhibition of v-ATPase proton pumping. Increasing lysosomal pH reduces the activity of LAL. The result of this is a time dependent switch of lysosomal accumulation of FC to CE. Early stage atherosclerosis may involve normal and perhaps increased LAL activity leading to cytosolic lipid droplet accumulation, whereas later stages of atherosclerosis may have an acquired dysfunction in LAL hydrolytic activity leading to lysosomal lipid sequestration. Therefore, later stages of atherosclerosis, where LAL function is inhibited, may be considered to be morphologically similar to Wolman disease and CESD, albeit for an entirely different reason.

Outside of alleviating premature onset of atherosclerosis associated with genetic deficiency in LAL, it is hard to predict whether augmentation of LAL is a good therapeutic strategy. There are indications that selective upregulation of the autophagic-lysosomal pathway may be able to recover lysosomal function and LAL hydrolysis. It is unclear, however, whether induction of this pathway beyond normal response is a viable *in vivo* strategy. Further research is necessary to know whether manipulation of arterial cell LAL activity is likely to alleviate or aggravate the consequences of cholesterol accumulation in the artery wall.

### Conflict of interest statement

The authors declare that the research was conducted in the absence of any commercial or financial relationships that could be construed as a potential conflict of interest.

## Abbreviations

ABCA1: ATP-binding cassette transporter A1
ACAT: acyl-coenzyme A:cholesterol acyltransferase
aggLDL: aggregated low density lipoprotein
apoA1: apolipoprotein A1
apoB: apolipoprotein B
CE: cholesteryl esters
CESD: cholesteryl ester storage disease
ER: endoplasmic reticulum
FC: free cholesterol
HDL: high density lipoprotein
LAL: lysosomal acid lipase
LDL: low density lipoprotein
LDL-R: low density lipoprotein receptor
nCEH: neutral cholesteryl ester hydrolase
NPC: Niemann Pick type C
oxLDL: oxidized low density lipoprotein
rhLAL: recombinant human lysosomal acid lipase
SMC: smooth muscle cell
TFEB: transcription factor EB
v-ATPase: vacuolar H^+^-ATPase
VLDL: very low density lipoprotein.
